# *π*‐Conjugation Induced Anchoring of Ferrocene on Graphdiyne Enable Shuttle‐Free Redox Mediation in Lithium‐Oxygen Batteries

**DOI:** 10.1002/advs.202103964

**Published:** 2021-11-25

**Authors:** Xudong Li, Guokang Han, Zhengyi Qian, Qingsong Liu, Zhuomin Qiang, Yajie Song, Hua Huo, Chunyu Du, Shuaifeng Lou, Geping Yin

**Affiliations:** ^1^ MIIT Key Laboratory of Critical Materials Technology for New Energy Conversion and Storage School of Chemistry and Chemical Engineering Harbin Institute of Technology Harbin 150001 P. R. China

**Keywords:** electron interactions, graphdiyne, lithium‐oxygen batteries, redox mediators, shuttle phenomena

## Abstract

Soluble redox mediators (RMs), an alternative to conventional solid catalysts, have been considered an effective countermeasure to ameliorate sluggish kinetics in the cathode of a lithium–oxygen battery recently. Nevertheless, the high mobility of RMs leads to serious redox shuttling, which induces an undesired lithium‐metal degeneration and RM decomposition during trade‐off catalysis against the sustainable operation of batteries. Here, a novel carbon family of graphdiyne matrix is first proposed to decouple the charge‐carrying redox property of ferrocene and the shuttle effects. It is demonstrated that a ferrocene‐anchored graphdiyne framework can function as stationary RM, not only preserving the redox‐mediating capability of ferrocene, but also promoting the local orientated three‐dimensional (3D) growth of Li_2_O_2_. As a result, the RM‐assisted catalysis in lithium–oxygen battery remains of remarkable efficiency and stability without the depletion of oxidized RMs or lithium degradation, resulting in a significantly enhanced electrochemical performance.

## Graphical abstract

Ferrocene‐anchored graphdiyne framework decouples the charge‐carrying redox property of ferrocene‐redox mediators and the shuttle effects, suppressing the consumption of oxidized ferrocene and degradation of the Li‐metal anode. Such ferrocene‐assisted catalysis results in a significantly enhanced electrochemical performance of Li—O_2_ batteries.

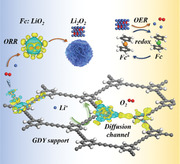

## 1 Introduction

Lithium–oxygen batteries (LOBs), owning the highest energy density among all battery systems, have been demonstrated as ultimate solutions to meet the urgent demand for high energy storage systems.^[^ ^1^, ^2^, ^3^ ^]^ Actually, sluggish kinetics and parasitic reactions are intractable hurdles in the practical reaction process of LOBs, especially in the air cathodes due to complex oxygen / lithium peroxide (O_2_/Li_2_O_2_) redox chemistry.^[^ ^4^ ^]^ In view of the current state‐of‐the‐art level of LOBs, scarce research is still focusing on maximizing specific capacity, but is replaced by ameliorating the energy efficiency and cycling stability.^[^ ^5^ ^]^ Various kinds of solid catalysts to date have been proposed to accelerate cathodic kinetics.^[^ ^6^, ^7^, ^8^ ^]^ However, the solid–solid catalysis acts only at the near surface of solid catalyst, which limits the reversible decomposition of Li_2_O_2_ and even leads to undesirable parasitic reactions involving the electrode and electrolyte.^[^ ^9^ ^]^

Soluble redox mediators (RMs), an emerging substitute to improve the rigid cathode electrochemistry, have been demonstrated as high efficiencies in decreasing the charge polarization and enhancing energy efficiency very recently.^[^ ^10^, ^11^ ^]^ Unlike the solid catalyst with area limitations of solid–solid contact, the soluble RMs serving as an electron–hole transfer agent can diffuse to anywhere of the cathode surface and reaction products. That transforms the catalytic dynamics from the solid–solid inhomogeneous type in the traditional catalyst to the solid–liquid type in the RMs catalyst, enlarging the reaction region and improving the catalytic functionality. Despite numerous superiorities, the applications of RMs for highly efficient cathode kinetics are yet limited by some awkward issues.^[^ ^12^ ^]^ First, the soluble nature of RMs in the electrolyte usually brings significant “shuttle effects” between the cathode and lithium anode, well known in lithium sulfur batteries, which further triggers an aggravated self‐discharge effect in the LOBs.^[^ ^13^ ^]^ Correspondingly, the battery life is significantly decreased due to the deterioration of Li anode and functional depletion of RMs. Second, the viscous resistance from the electrolyte obviously reduces the transfer efficiency of RM, especially given the shuttle catalysis of RM between electrolyte and cathode.^[^ ^14^ ^]^

Practically, immobilizing RMs on the cathode side is a direct and effective way to solve the aforesaid issues. However, little attention has been paid to this key point so far. Limited attempts on the RM immobilization are based on the physical combination between organic catalyst and conductive polymer film,^[^ ^15^ ^]^ yet it is obviously unsuitable for the long‐term and stable LOBs due to the contact loss between RM and support. Developing a practicable RM immobilization strategy to establish a robust RM‐support framework emerging as an ideal project to boost the practical applications of LOBs. Metallocene molecules (e.g., ferrocene, cobaltocene, nickelocene, etc.), a type of representative RM, have been demonstrated to promote oxygen reduction reaction (ORR) and oxygen evolution reaction (OER) kinetics in discharge/charge process.^[^ ^16^, ^17^ ^]^ Interestingly, these molecules contain a *π*‐electron system in cyclopentadienyl rings, which can be intercalated in layered carbon hosts through the *π*–*π* stacking.^[^ ^18^, ^19^ ^]^ Therefore, it is a possibility to utilize this confinement effect from the *π*‐ conjugation system to immobilize metallocene molecules to construct a stabilized RM‐support framework.

Graphdiyne (GDY), with a *π*‐conjugated network of sp‐ and sp^2^‐hybridized carbon atoms,^[^ ^20^ ^]^ is probably a target support to couple and immobilize metallocene molecules. Compared with the prevailing sp^2^‐hybridized carbon materials, the uneven distribution of electrons and high controllability in physical and chemical properties for GDY makes it more suitable to the construction of highly efficient cathode substrate. On the one hand, the sp‐hybridized carbon atoms can easily bind O_2_ due to the electron deficiency property based on the Mulliken charge density principle, which largely facilitates OER kinetics. On the other hand, the topological GDY with larger hexagon‐shaped pores completely ensures efficient electron and mass transfer during cathodic catalysis. Despite these natural advantages, there have been no reports regarding the GDY as cathodic framework in the LOBs up to now.

For the first time, we tailored the GDY matrix to immobilize ferrocene (Fc) and decoupled the charge‐carrying redox property of Fc redox mediators and the shuttle effects. Benefitting from that, the Fc‐assisted catalysis from the original charge carrying by physical migration in the electrolyte is transformed into charge carrying by electron transfer on air electrode while retaining the redox‐mediating capability, suppressing the consumption of oxidized Fc and degradation of the Li‐metal anode. In addition, owing to the electronic interaction between the GDY and Fc, the originally ORR‐inactive Fc was activated and therefore promoted local orientated 3D‐growth of Li_2_O_2_, which alleviates the “sudden death” of LOBs. In this work, we especially emphasize the significance of cathode construction design and effects of the electronic RM‐support interaction on catalytic activity. It provides a new insight into the sustainable employments of redox mediators and the design principles of novel electrode for advanced electrocatalysis in LOBs.

## 2 Results and Discussion

### 2.1 Characterization of GDY and GDY/Fc

**Figure 1:**
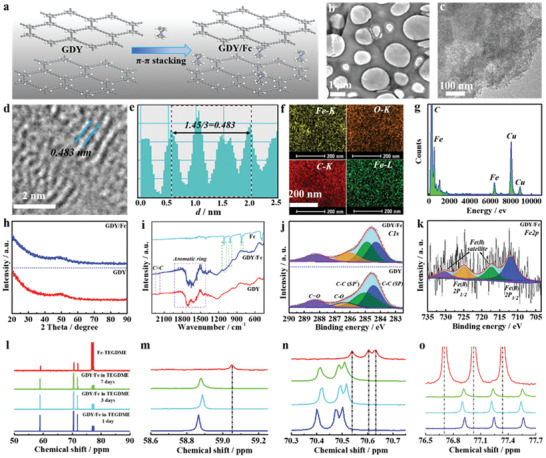
a) Schematic illustration of synthesis of the GDY/Fc. b,c) TEM images, d) HRTEM image (blue lines signify the lattice spacing), e) profile of the interlayer distance, f) corresponding elemental mapping, g) EDX spectrum of the GDY/Fc, h) XRD patterns, and i) FTIR spectra of the GDY/Fc, respectively. j) High‐resolution C 1s XPS spectra of the GDY and GDY/Fc. k) High‐resolution Fe 2p XPS spectrum of the GDY/Fc. l–o) Time‐resolved 13C NMR spectra of TEGDME with GDY/Fc electrode.

At first, we would like to make a fundamental study on the structure and chemistry of the RM‐anchored catalysts. 2D layered GDY with a delocalized *π*‐system and larger hexagon‐shaped pores (diameter of ca. 16.3Å, Figure S1, Supporting Information) was tailored as the support matrix (Figures S2 and S3, Supporting Information). By a robust *π*–*π* interaction, the Fc molecules were immobilized on GDY non‐covalently (GDY/Fc), as illustrated in **Figure** **1**a. Transmission electron microscopy (TEM) imaging (Figure 1b,c) and high‐resolution TEM (HRTEM, Figure 1d) show a well‐defined 2D layered structure of GDY with interlayer spacing of 0.483 nm (Figure 1e), where Fc molecules (7.04 wt.%,) are uniformly dispersed on the layered GDY (Figure 1f,g). No diffraction peaks for Fc appear in the X‐ray diffraction (XRD) pattern (Figure 1h), excluding formation of Fc clusters. The Fourier transform infrared spectroscopy (FTIR, Figure 1i), X‐ray photoelectron spectroscopy (XPS, Figure 1j,k), and N_2_ adsorption–desorption analysis (Figure S4, Supporting Information) further confirm the Fc has been incorporated into GDY while maintaining its pristine molecular composition and structure. In time‐resolved 13C nuclear magnetic resonance spectra (NMR, Figure 1l–o), no signals of Fc were detected in the electrolyte with GDY/Fc, even after a 7‐day immersion (Figure S5, Supporting Information), which clearly indicates indissolubility of the anchored Fc and robust *π*–*π* interaction between Fc and GDY support.

### 2.2 Redox Kinetics of Fc and Electrochemical Behaviors of GDY/Fc

**Figure 2:**
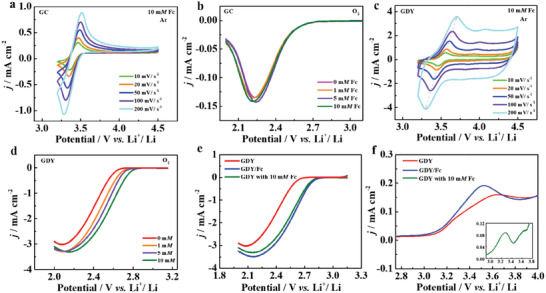
a) Cyclic voltammetry curves of GC with 10 mm Fc under Ar atmosphere at various scan rates. b) ORR polarization curves of GC with different Fc concentrations. c) Cyclic voltammetry curves of GDY with 10 mm Fc under Ar atmosphere at various scan rates. d) ORR polarization curves of GDY with different Fc concentrations. e) ORR and f) OER polarization curves of GDY, GDY/Fc, GDY with 10 mm Fc, respectively.

Prior to evaluating the immobilized RM of Fc, we examined the redox properties of soluble Fc on glassy carbon electrodes (GC) by rotating disk electrode (RDE) technique. The CV results in Ar‐saturated electrolyte (**Figure** **2**a) verify that the redox potential (3.46‐3.51 V) of Fc is higher than the theoretical redox potential (2.96 V) of O_2_/Li_2_O_2_, suggesting that Fc can serve as an efficient RM to facilitate the oxidation of Li_2_O_2_. In O_2_‐saturated electrolyte (Figure 2b), the ORR cathodic peak currents and onset potential keep nearly constant with the increased Fc concentration, indicating no positive role of soluble Fc during the ORR process. However, we observed that the ORR properties of Fc on GDY‐coated GC electrode are quite different from that on GC electrode. Despite the similar couple of redox peaks in CV measures (Figure 2c), the ORR onset potentials rise visibly as Fc concentration increases. We, therefore, suspect that the GDY support activates originally ORR‐inactive Fc to active site for ORR process.

In fact, given that the *π*–*π* interaction between the layered GDY and ferrocene, more Fc in electrolyte may be absorbed on GDY with increase of Fc concentration.^[^ ^21^ ^]^ We hold the opinion that the electronic interaction in RM‐support is crucial to the synergistic enhancement of electrocatalytic performance. To demonstrate the OER behavior, we conducted chronopotentiometry (CP) in advance to deposit equal amounts of Li_2_O_2_ (Figure S6, Supporting Information), and then sweeping in an OER involved potential. Similarly, the polarization currents increase significantly as more Fc introduced in electrolyte during OER process (Figure S7, Supporting Information). We conclude that, collectively, Fc can work effectively on GDY support in both OER and ORR process. The results well support our achievements in the establishment of efficient GDY‐Fc systems in LOBs.

To better understand the effectiveness of the immobilized Fc on GDY, we compared the electrochemical performances of GDY/Fc with pristine GDY that in the absence/presence of soluble Fc. Based on the ORR curves (Figure 2d,e), a much higher ORR current with a prior onset potential is detected on the GDY/Fc electrode than those on the pristine GDY electrodes, suggesting that GDY/Fc can effectively accelerate the ORR kinetic and produce more Li_2_O_2_. In addition, the GDY/Fc electrode delivers a much higher OER current with a lower onset potential (Figure 2f and Figure S8, Supporting Information), indicating the enhanced Fc‐assisted decomposition of Li_2_O_2_. To summarize, the Fc anchored on GDY support can not only modulate ORR activity, but also retain its charge‐carrying redox activity.

### 2.3 Performances of LOBs with Different Cathodes

**Figure 3:**
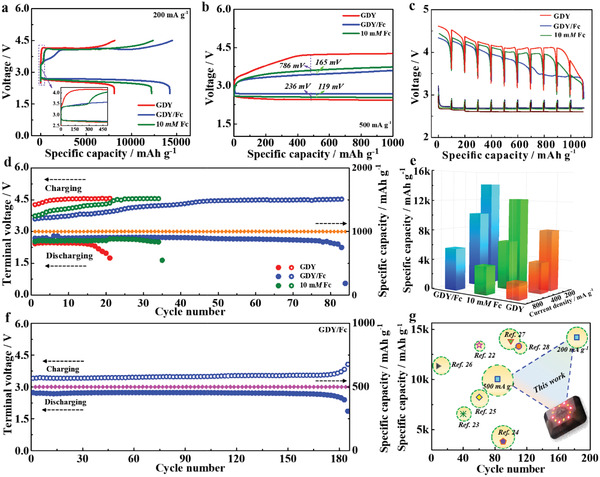
Li—O_2_ cells performance with GDY, GDY/Fc, and GDY with 10 mm Fc. a) Galvanostatic discharge/charge curves at a current density of 200 mA g^−1^ between 2.0 and 4.5 V, and b) Galvanostatic discharge/charge curves at a current density of 500 mA g^−1^ under an upper‐limit capacity of 1000 mA h g^−1^. c) GITT curves at a fixed current density of 200 mA g^−1^ with a relaxation time of 6 h. d) Cyclic performance at a current density of 500 mA g^−1^ with a capacity limitation of 1000 mA h g^−1^. e) Capacities at various current densities. f) Cyclic performance of Li—O_2_ cell with GDY/Fc at a current density of 200 mA g^−1^ under a capacity limitation of 500 mA h g^−1^. g) Performance comparison for other representative published efforts. The data includes following parameters: cycle number (*x* axis), specific capacity (*y* axis), and current density (circle radius).

Inspired by the highlighted electrocatalysis dynamics of GDY/Fc electrode, we would like to evaluate the promising applications of GDY/Fc as cathode in Li—O_2_ cell. As shown in **Figure** **3**a, the cell with GDY/Fc electrode exhibits an impressive discharge specific capacity of 14231 mA h g^−1^ at a current density of 200 mA g^−1^, while the capacity is only 4922 mA h g^−1^ for the case with ketjen black (KB, Figure S9, Supporting Information), suggesting that the capacity of the Li—O_2_ cell is mainly contributed by GDY‐based catalysts rather than KB. Noteworthy is that the discharge capacity of LOBs with GDY/Fc rises visibly as Fc immobilized amount increases (Figure S10, Supporting Information), indicating the enhancement effect of immobilized Fc on LOB performance. In comparison with the cells with GDY (and with 10 mm Fc), the cell with GDY/Fc retains higher discharge capacity of 10 071 and 5613 mA h g^−1^, even at a current density of 400 and 800 mA g^−1^ (Figure 3e and Figure S11, Supporting Information), respectively. More remarkably, the cell with GDY/Fc presents a lowered discharge–charge polarization (Figure 3b), especially OER overpotential, indicating that the Fc anchored on GDY still retain its charge‐carrying redox capability, leading to greatly improved round‐trip efficiency. In galvanostatic intermittent titration technique (GITT) measurements, the cell with GDY/Fc exhibits much smaller gap between the thermodynamic equilibrium potential and the discharge–charge plateau (Figure 3c), implying a small polarization and low energy barrier in the actual electrocatalytic process.

As observed in Figure 3d and Figure S12a–c, Supporting Information, the cell with GDY/Fc maintains a long cycle lifetime of 83 cycles at a high current density of 500 mA g^−1^ with a capacity limitation of 1000 mA h g^−1^, 4.3 (and 2.5) times higher than the lifespan of the cell with GDY (and with 10 mm Fc). Moreover, the cell with GDY/Fc can normally run for 183 cycles at a current density of 200 mA g^−1^ with a capacity limitation of 500 mA h g^−1^ (Figure 3f and Figure S12d, Supporting Information), and 85 cycles even at 2000 mA g^−1^ with an upper‐limit capacity of 2000 mA h g^−1^ (Figure S13, Supporting Information), respectively. To our knowledge, those performance parameters of the cell with GDY/Fc were superior to that of traditional catalysts reported in previous literatures (Figure 3g and Table S1, Supporting Information).^[^ ^22^, ^23^, ^24^, ^25^, ^26^, ^27^, ^28^ ^]^ Herein a key question emerging and puzzles us, what caused the enhancement of capability and cycle life for LOB with GDY/Fc. To gain more fundamental insight into it, it is indispensable to investigate the corresponding status of those cathodes and Li metals anode during the persistent discharge–charge process.

### 2.4 Evolution of Cathodes Products during Discharge and Charge Process

**Figure 4:**
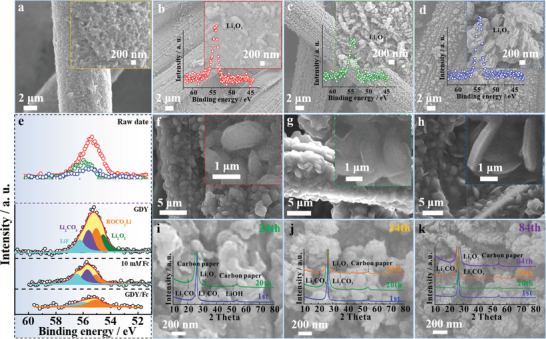
SEM images of a) pristine GDY/Fc electrode, and the cathodes after discharged to 1000 mA h g^−1^ with b) GDY, c) GDY in the presence of 10 mm Fc, and d) GDY/Fc, Insets are the corresponding Li 1s XPS spectra. e) Original (top) and corresponding fitted Li 1s XPS (bottom) spectra of the cathodes after recharge. SEM images of the cathodes after full discharge with f) GDY, g) GDY in the presence of 10 mm Fc, and h) GDY/Fc. SEM images of the cathodes after long cycling with i) GDY (20th), j) GDY in the presence of 10 mm Fc (34th), and k) GDY/Fc (84th), insets are the corresponding XRD at different cycle numbers.

The evolution of cathodes products at different discharge‐recharge stages was investigated. **Figure** **4**a and Figure S14, Supporting Information, exhibits the morphology of these pristine electrodes. After discharging to the limited capacity of 1000 mA h g^−1^, products with different morphologies of toroidal, peas and petal are homogeneously distributed on the GDY (without soluble Fc), GDY with 10 mm Fc and GDY/Fc cathodes (Figure 4b–d and Figure S15a–i, Supporting Information), respectively, which can be identified as Li_2_O_2_ by high‐resolution XPS analysis (inset of Figure 4b–d).^[^ ^29^ ^]^ The observation is that more ordered, loosened and larger micron‐sized Li_2_O_2_ particles on the GDY/Fc‐catalyzed electrode is of note, which may be relevant to the local orientated 3D‐growth of Li_2_O_2_ rather than a compact film‐growth character. Under this model, the uniformly and loosely distributed 3D‐Li_2_O_2_ can not only enhance the charge/mass transfer in the gas‐Li_2_O_2_‐electrolyte three‐phase interface, but also promote the efficient transfer of electron in the inner of Li_2_O_2_, thereby enhancing reaction kinetics and retarding the fast passivation of O_2_‐electrode. As revealed in Li 1s XPS spectra (Figure 4e), Li_2_O_2_ petals on the GDY/Fc cathode were almost decomposed completely after the subsequent recharge, which is consistent with our inference. In combination with the complementary electrochemical impedance spectra (EIS, Figure S16 and Table S2, Supporting Information), it is reasonable to deduce that the Fc‐incorporated cathode is capable of promoting the reversible formation and decomposition of Li_2_O_2_. The chemical interactions between Fc and Li_2_O_2_ were further identified by ex‐situ XPS (Figure S17, Supporting Information), as evidenced by the higher shift of binding energies for Fe 2p components upon Li_2_O_2_ adsorption. At the end of discharge, we observed the formation of small sized toroidal with high packing density on the GDYs surface (Figure 4f,g). The produced Li_2_O_2_ at the cell with GDY/Fc, however, presents coin‐shaped morphology with large size of ≈3 µm in diameters (Figure 4h), further confirming the orientated growth of Li_2_O_2_.^[^ ^15^ ^]^

To evaluate the reversibility of three kinds of cathodes, the corresponding O_2_‐electrodes prepared from several charged cells extracted following different cycle numbers were characterized. XRD (inset of Figure 4i,j) and Raman patterns (Figure S18a,b, Supporting Information) reveal the formation of Li_2_CO_3_ and LiOH as main byproducts on cathodes with GDYs after 20th cycle.^[^ ^30^ ^]^ In stark contrast, there are no signals of Li_2_CO_3_ and LiOH can be detected on GDY/Fc electrode (inset of Figure 4k and Figure S18c, Supporting Information), even after 50th cycle. These results evidence that the GDY/Fc catalyst can minimize side reactions involving the O_2_‐electrode and electrolyte to improve the durability of LOB. After long cycling, we observed that the rock‐like products (including Li_2_O_2_, Li_2_CO_3_, LiOH, Li_2_RCO_3_, LiAc, et al.) are closely stacked on the surface of cathodes with GDYs (Figure 4i,j). This severe passivation of O_2_‐electrodes causes the “sudden death” of LOBs. In comparison, the accumulative products on the GDY/Fc‐catalyzed electrode still take on a petaloid structure (Figure 4k), demonstrating the excellent reversibility of GDY/Fc. With the help of ex‐situ XPS, knowledge of the stability of the Fc on GDY in the long‐term cycling was also obtained. It is noted that the Fe 2p spectra of the cycled GDY/Fe electrodes (Figure S19, Supporting Information) bear resemblance to that of the pristine electrodes with a slightly shift of Fe 2p3/2 and Fe 2p1/2, implying no obvious degradation of the immobilized Fc molecule during the repeated cycling. Moreover, in situ differential electrochemical mass spectrometry (DEMS) procedures were conducted to monitor the evolved gases on GDY/Fc cathode during charging in the 1st, 10th, and 100th cycles. The DEMS curves (Figure S20, Support information) confirm the oxidation processes have been overwhelmingly governed by oxygen releasement. The corresponding ratios of *v*(e^−^):*v*(O_2_) were calculated as about 2:1, 2.01:1, and 2.43:1, respectively, based on the Equation S1, Supporting Information,^[^ ^31^ ^]^ within the tolerance of permitted error despite the fluctuation, further demonstrating considerable reversibility of Li_2_O_2_ formation/decomposition on GDY/Fc cathode.^[^ ^32^ ^]^ Overall, the efficiency of Fc‐mediated Li_2_O_2_ kinetics demonstrates remarkably stable without the consumption of Fc, leading to an improved performance of LOB.

### 2.5 Analysis of the Li Metal Anodes after Cycling

**Figure 5:**
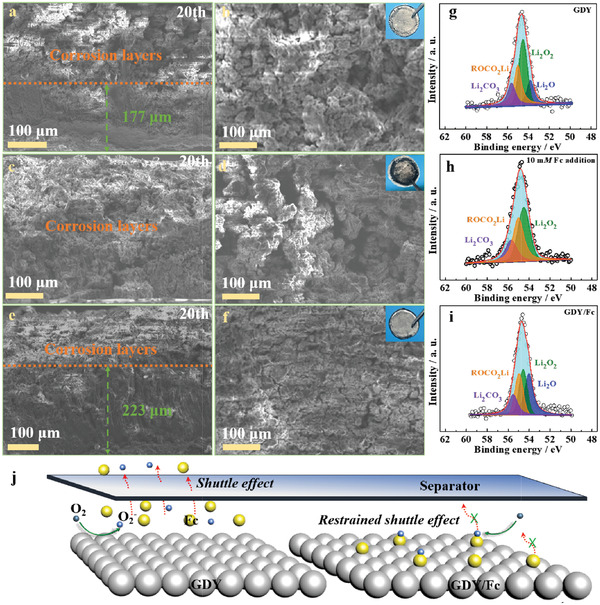
Cross‐sectional and surface SEM images of the Li mental anodes after 20th cycles with a,b) GDY, c,d) GDY in the presence of 10 mm Fc, and e,f) GDY/Fc. The corresponding Li 1s XPS spectra with g) GDY, h) GDY in the presence of 10 mm Fc, and i) GDY/Fc. j) A schematic illustration of the Li protection for the GDY/Fc cathode.

To verify the protective effect of the RM molecular‐confining catalytic strategy on Li metal anode, especially its ability to alleviate side reactions caused by shuttle effect, we further analyzed Li metal anodes after cycling. **Figure** **5**a–f shows the overall scanning electron microscopy (SEM) images of the Li metal anodes taken from the charged cells with different cathodes after 20 cycles. The Li metal anodes with GDY (Figure 5a,b) present serious corrosion layers upon cycling, and the corrosion grade apparently becomes more and more severe as the 10 mm Fc introduced, leaving much corrosion hole on the surface (Figure 5c,d). Encouragingly, the cycled Li metal anode with GDY/Fc maintains extremely high morphological completeness, with the highest bulk Li metal retention (Figure 5e,f). It is worth noting that the compositions of the corrosion layers on Li metal (Figure 5g–i) mainly contain Li_2_CO_3_, ROCO_2_Li, Li_2_O_2_ and Li_2_O (considering Li self‐oxidation in testing process).^[^ ^33^ ^]^ The cause of corrosion can be ascribed to i) the reactivity of Li metal with the free O_2_^−^‐intermediates and solvent molecules in electrolyte, producing Li_2_O_2_, Li_2_CO_3_, ROCO_2_Li; and ii) oxidized Fc (Fc^+^) shuttles to the anode, aggravating Li anode corrosion and producing more Li_2_O_2_. We therefore speculate that the enhanced stability of Li metal anodes observed here can be attributed to the positive effect on cathode with GDY/Fc, which effectively eliminates the shuttle effect to against the attacks of superoxide radical anions and Fc^+^ on the active Li metal anode (schematic diagram in Figure 5j).

### 2.6 Theoretical Simulation and Mechanism Analysis

**Figure 6:**
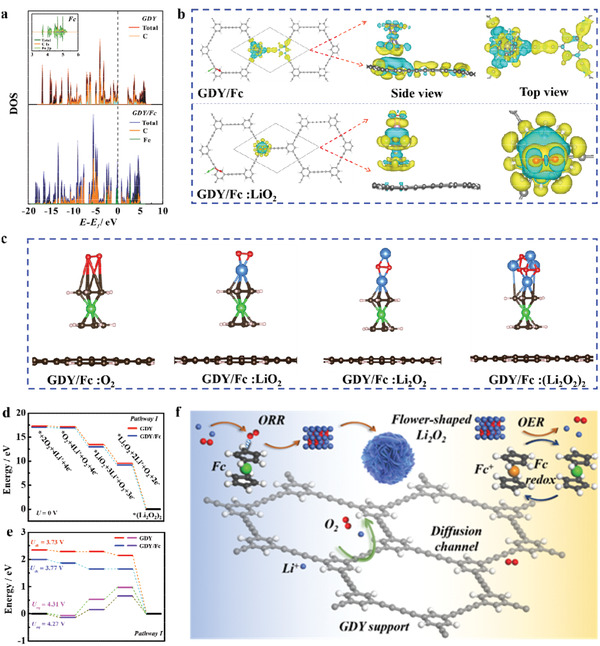
a) DOS map of the GDY, Fc, and GDY/Fc. b) Optimized structure of GDY/Fc and GDY/Fc:LiO_2_, and corresponding charge density distribution (the blue area and yellow area stand for the lost electron and the gained electron, respectively). c) Optimized structures of O_2_, LiO_2_, Li_2_O_2_, and (Li_2_O_2_)_2_ adsorbed on GDY/Fc. d,e) Calculated free energy diagrams for the discharge reactions on the active surface of GDY (redline) and GDY/Fc (blue line). f) A schematic illustration of the ORR processes occurring on the GDY/Fc cathode during discharge in Li—O_2_ battery.

Density functional theory (DFT) calculations were performed to unravel the in‐depth electronic RM‐support interaction and the underlying reaction mechanism in discharge kinetics. The projected density of states (PDOS, **Figure** **6**a) reveal the evolution of electron orbitals between Fc and GDY. Compared with isolated Fc, the *d* band of Fe in GDY/Fc decreases with the introducing of GDY support, which triggers higher state density around Fermi level.^[^ ^34^ ^]^ The corresponding charge density deference diagrams (the top of Figure 6b) show abundant electron transfers at the GDY/Fc interface, manifesting the strong electronic interaction between Fc and GDY. It is noted that Fc presents electron‐deficiency property after being anchored on GDY support, which may lead to enhancement of the adsorption capacity for GDY/Fc on intermediates species. It's precisely the originally ORR‐inactive Fc was activated to predominant active catalytic sites for ORR due to the change of electronic structure and charge redistribution on GDY/Fc, which is evidenced by the stable adsorption of LiO_2_ on GDY/Fc and accompanied apparent electrical coupling (the bottom of Figure 6b).

Based on different reaction paths, we demonstrated the calculated free energy pathways toward discharge process of O_2_ cathode with GDY and GDY/Fc. Figure 6c presents the optimized structures of O_2_, LiO_2_, Li_2_O_2_, and (Li_2_O_2_)_2_ adsorbed on GDY/Fc, and the corresponding pathway I is shown here (Equations (1)–(4) and Figure 6d,e) and the pathway II is shown in Equation S2 and Figure S21, Supporting Information.

(1) $$O_{2}\;+\;e^{-}\;+\;\mathrm{GDY}/\mathrm{Fc}\;\to {\;\mathrm{GDY}/\mathrm{Fc}:\;O}_{2\;}^{-}$$

(2) $${\mathrm{GDY}/\mathrm{Fc}:\;O}_{2\;}^{-}\;+\;Li^{+}\;\to \;\mathrm{GDY}/\mathrm{Fc}:\;\mathrm{Li}O_{2}$$

(3) $$\mathrm{GDY}/\mathrm{Fc}:\;\mathrm{Li}O_{2}\;+\;Li^{+}+\;e^{-}\;\to \;\mathrm{GDY}/\mathrm{Fc}:\;Li_{2}O_{2}$$

(4) $$\mathrm{GDY}/\mathrm{Fc}:\;Li_{2}O_{2}+O_{2}+2Li^{+}+2e^{-}\to \mathrm{GDY}/\mathrm{Fc}:{\left(Li_{2}O_{2}\right)}_{2}$$

The calculated results reveal that the Δ*G* for the most endothermic step through pathway I is smaller than that through pathway II on both models (Table S3, Supporting Information), thus it is evident that the pathway I plays a leading role in ORR process. To be specific, the GDY/Fc first capture the dissociative O_2_^−^ to form a complex of GDY/Fc:O_2_^−^ during discharge process, which will bond Li^+^ turning to GDY/Fc:LiO_2_ by subsequent single electron transfer reaction, and eventually convert to GDY/Fc:(Li_2_O_2_)_2_ via further reduction reaction. It turns out that the calculated discharge overpotential for GDY/Fc is 0.5 V, 80 mV higher than that of the GDY (0.58 V), indicating that the immobilized Fc active sites on GDY can generate significant effect on enhancing ORR kinetics, which is strongly corroborated in the obtained experimental observations. Here, we found that the binding energy of the intermediates (O_2_^−^ and LiO_2_) at GDY/Fc is much higher than that on GDY (Figure S22, Supporting Information), which favors the shuttle suppression for these active species and the local orientated growth of Li_2_O_2_ on the Fc site. The DFT supports a fact that the immobilized Fc on GDY support plays a pivotal role in boosting ORR process. In addition, we believe that the Fc with charge‐carrying redox activity can chemically decompose Li_2_O_2_ more efficiently during charge process owing to the immediate contact between Fc and Li_2_O_2_, which enables a fast recover of the active sites the GDY/Fc.^[^ ^14^ ^]^ Based on the experimental results and mechanism analysis, a schematic illustration of the ORR/OER processes occurring on the GDY/Fc catalyst is demonstrated in Figure 6f.

## 3 Conclusion

In this work, we tailored an ideal GDY cathode support for metallocene (e.g., Fc) to decouple the charge‐carrying redox property of RM and the shuttle effects. The immobilized Fc on the GDY frame can not only promote favorable ORR/OER kinetics, but also effectively suppress the consumption of oxidized Fc and degradation of the Li‐metal anode. Beyond our expectations, the GDY support activates originally ORR‐inactive Fc to active site. With the GDY/Fc, its firm ability to capture O_2_^−^ and LiO_2_ promotes local orientated 3D‐growth of Li_2_O_2_ and guard against the reactive intermediate‐triggered parasitic reactions. Moreover, the charge‐carrying redox property of Fc can trigger a chemical decomposition of Li_2_O_2_, which enables a fast recover of the active sites. Benefiting from such Fc‐assisted catalysis, the LOB with GDY/Fc delivers a high capacity of 14231 mA h g^−1^ and high stability over 183 cycles.

From a practical point of view, considering the severe issues of shuttle effect and poor mass‐transfer efficiency of mobile RM, it is vital significance to anchor RM on the cathode side for the advancement of LOBs. Herein, in addition the design of RM‐anchored cathode, we particularly discussed RM‐support interaction. Making full use of the electronic interaction between RM and support is one of the most significant strategies to synergistically promoting the electrocatalytic performance. Despite of no Li‐protective layers or functional separators, our work suggests that the Fc loading on GDY support can effective decouple the charge‐carrying redox property of RM and the shuttle effects, which opens a new pathway for the sustainable employments of RMs in LOBs.

## 4 Experimental Section

### Synthesis of Graphdiyne Powder

GDY was synthesized in accordance with reference as reported,^[^ ^20^ ^]^ but with some modifications here. Cleaned copper foils (size of 14 × 14 mm, 16pc) were added into 50 mL pyridine solution, and stirred at 60 °C in the water bath, while 25 mL pyridine solution of 10 mg 1,3,5‐triethynylbenzene was added with a peristaltic pump for 4 h, before the reaction vessel was transferred to an oven at 60 °C and left unstirred for 3 days under an absolute dark condition. Then those treated copper foils were taken out and cleaned using acetone, *N*‐methyl pyrrolidone (NMP), ethanol, hydrochloric acid solution, and water, sequentially, to yield yellowish GDY films. After that, the GDY films were processed by ultrasonic treatment for 2 h and immersed in concentrated nitric acid for 10 h, successively. Finally, the product was washed with purified water and subsequently freeze‐dried to obtain GDY powder.

### Synthesis of Graphdiyne/Ferrocene Catalysts

The as‐synthesized GDY powder (10 mg) was dispersed in 3 mL ethanol by sonication for 2 h then 5 mL ethanol solution of ferrocene (100 mg) was added into the former dispersion drop by drop. After stirring for 48 h, the suspension was filtrated and freeze dried to get the GDY/Fc sample.

### Preparation of Thin‐Film Working Electrode

To prepare the GDY‐based catalyst ink, 2.2 of mg GDY‐based catalyst was ultrasonically dispersed in a solution of 30 µL Nafion (5 wt%) and 520 µL mixture (isopropanol/water = 1:3). The catalyst ink was cast onto a polished glassy carbon (GC) RDE (*d* = 3 mm), forming the thin‐film working electrode. The catalyst loadings on RDE were 0.4 mg cm^−2^.

### RDE Measurements

RDE electrochemical tests were performed on an electrochemical workstation in a standard three‐electrode cell. 1 m LiTFSI in TEGDME solution was used as electrolyte. A platinum plate was used as the counter electrode, an Ag/Ag^+^ electrode was used as a reference electrodeafter calibration (*V* = 3.43 V vs. Li/Li^+^). The potentials are presented with respect to the Li/Li^+^ reference electrode. Prior to each experiment, the electrolyte was purged with Ar or O_2_ for 30 min to remove interference. Cyclic voltammetry (CV) experiments were performed with a different scan rate between 3.2 and 4.5 V versus Li/Li^+^ at room temperature. The ORR/OER activities were evaluated by linear sweep voltammetry (LSV). Typically, for the ORR measurements, the catalyst‐coated GC electrode was pre‐treated by CV at a scanning rate of 10 mV s^−1^ with a rotating rate of 900 rpm at a potential region of 3.15–2 (3 cycles), 3.15–3 (5 cycles), and 3.15–2 V (2 cycles), successively, in electrolyte saturated with argon. After that, the electrode was cycled at voltage range of 3.15–3 V for 30 cycles before obtaining the ORR polarization curve by LSV from 3.15 to 2 V in oxygen‐saturated electrolyte. For the OER tests, chronopotentiometry (CP) was first conducted to deposit an equal amount of Li_2_O_2_ on different catalysts by conducting a cathodic current of 10^−5^ A for 900 s in oxygen‐saturated electrolyte, and the OER polarization curve can be obtained by LSV from 2 to 4.5 V at a scanning rate of 2 mV s^−1^. All the current densities presented in the figures were normalized to the geometry area of GC disk.

### Li—O_2_ Cell Assembly and Testing

The cathode catalysts (60%) were uniform mixed with KB (30%) and carboxymethylcellulose (CMC, 10%) in isopropanol to form a homogeneous ink, which was sprayed on a piece of carbon paper. After vacuum drying at 80 °C for 12 h, the electrodes were cut into circular pieces with a diameter of 14 mm obtaining the O_2_‐electrodes. The electrolyte was 1 m LiTFSI in TEGDME, the counter electrodes were lithium foils and separators were Whatman GF/D glass fibers. The 2032‐tyoe coin cells were assembled in an argon‐filled glovebox with oxygen and moisture contents below 0.1ppm. The galvanostatic charge‐discharge tests were performed on a Neware TC51 battery test system at different current densities in the voltage range of 2.0–4.5 V versus Li/Li^+^, or in an upper‐limited capacity. The long‐term cycling measures were examined at different current densities under different limited capacities. The galvanostatic intermittent titration technique (GITT) measurements were carried out at a fixed current density of 200 mA g^−1^ with a relaxation time of 6 h. The electrochemical impedance spectroscopy (EIS) was conducted on a CHI660c electrochemical workstation in a frequency range of 10 mHz to 100 kHz. The in situ DEMS procedures were conducted at a current density of 200 mA g^−1^ with a limited‐capacity of 500 mA h g^−1^. All the measures mentioned above were conducted at room temperature. Specific capacity and current density were normalized by the mass of the GDY‐based catalysts loaded on the cathode.

### Characterizations

Transmission/high‐resolution transmission electron microscope (TEM/HRTEM) images, energy‐dispersive spectrometry (EDS), and mapping images were conducted on a Tecnai G2F30 transmission electron microscope operated at 200 kV. X‐ray diffraction (XRD) patterns were performed on an Empyrean diffractometer with Cu K*α* radiation (*λ* = 1.54178 Å) over the range 20° to 90°. Fourier transform infrared spectroscopy (FTIR) spectra were obtained using a Nicolet iS10 spectrograph. XPS were collected on a PHI 5700 ESCA System X‐ray microprobe with a focused monochromatic Al K*α*. Inductively coupled plasma (ICP) was conducted on a PerkinElmer Optima 5300DV ICP‐OES System optical emission. SEM images were conducted on a Helios Nanolab600i scanning electron microscope with an accelerated voltage of 20 kV. Brunauer−Emmett−Teller and Barrett−Joyner−Halenda models were used to determine the specific surface area and pore size distribution by nitrogen sorption measurements performed at 77 K (Beishide).

### Computational Calculations

DFT calculations are performed using VASP with PAW pseudopotentials and the GGA/PBE function^[^ ^24^, ^25^ ^]^ (the exchange–correlation functional chosen is Generalized gradient approximation [GGA] of Perdew–Burke–Ernzerhof [PBE]). An energy cutoff of 400 eV and the gamma k‐point are used for all calculations. Symmetric supercells are composed of 1‐atomic‐layer‐thick slabs separated by 20 Å of vacuum perpendicular to the surface to prevent spurious interactions due to periodic boundary conditions. Structures are relaxed until the forces on each atom are less than 10–3 eV Å^−1^. The chemisorption energy was calculated as follows:

(5) $$E_{\mathrm{absorption}}\;=\;E-\;E_{a}\;-\;E_{b}$$

where E is the total energy of the adsorbed system, *E*_a_ and *E*_b_ represent the total energy of free species and bare surface, respectively.

## Conflict of Interest

The authors declare no conflict of interest.

## Supporting information

Supporting Information

## Data Availability

Research data are not shared.
