# Supplementary material for: π‐Conjugation Induced Anchoring of Ferrocene on Graphdiyne Enable Shuttle‐Free Redox Mediation in Lithium‐Oxygen Batteries
Source: Adv Sci (Weinh). 2021 Nov 25;9(4):2103964. doi: 10.1002/advs.202103964 (PMC8811833; doi:10.1002/advs.202103964)

© 2021 Wiley-VCH GmbH

ADVANCED SCIENCE
Open Access

Supporting Information

for *Adv. Sci.*, DOI: 10.1002/advs.202103964

π-conjugation Induced Anchoring of Ferrocene on Graphdiyne Enable Shuttle-Free Redox Mediation in Lithium-Oxygen Batteries

Xudong Li, Guokang Han, Zhengyi Qian, Qingsong Liu, Zhuomin Qiang, Yajie Song, Hua Huo, Chunyu Du, Shuaifeng Lou, Geping Yin**

Supporting Information

π-conjugation Induced Anchoring of Ferrocene on Graphdiyne Enable Shuttle-Free Redox Mediation in Lithium-Oxygen Batteries

Xudong Li, Guokang Han, Zhengyi Qian, Qingsong Liu, Zhuomin Qiang, Yajie Song, Hua Huo, Chunyu Du, Shuaifeng Lou, Geping Yin**

Figure S1 a) Structures geometry of GDY. b) Side view of GDY. c) 2D nanosheet with interlayer spacing of 0.483 nm for prepared GDY. d) Full atomistic model of GDY depicting the atomistic sexangular pores, with van der Waals openings on the order of 1.63 nm.

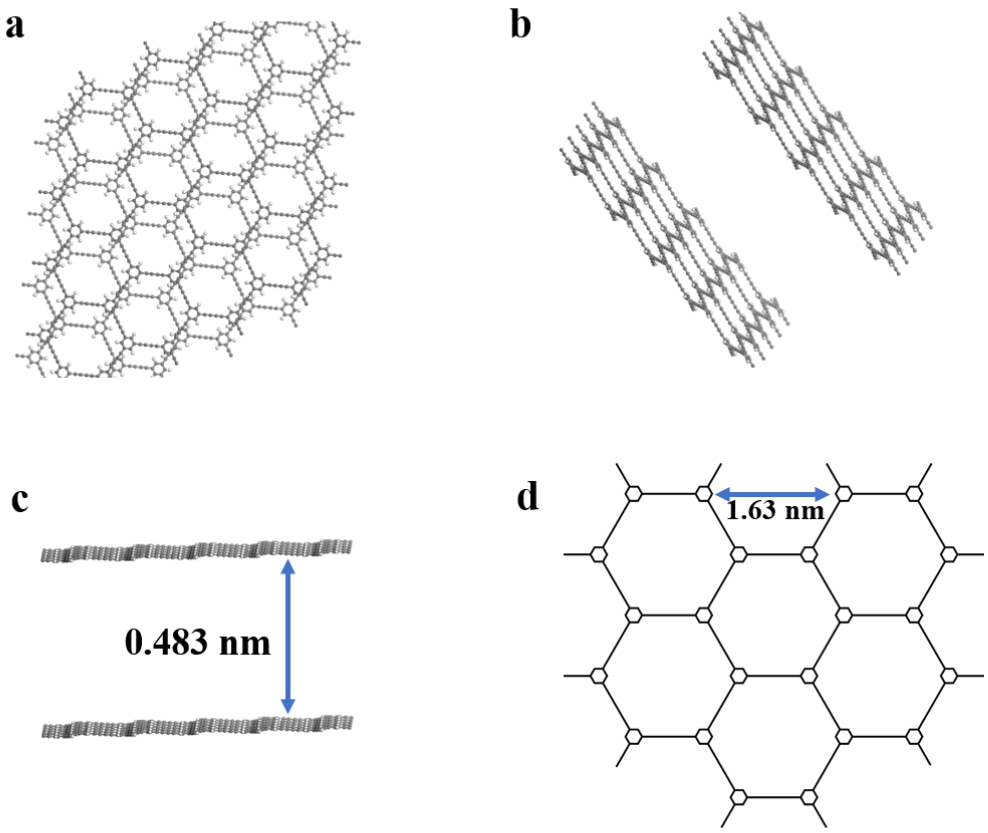

Figure S2 Digital photos of a) GDY and b) GDY/Fc.

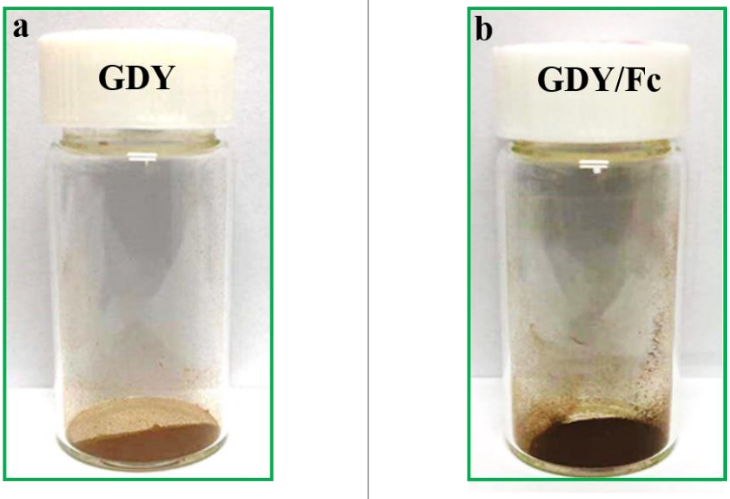

Figure S3 a) TEM images, b) Elemental mapping. c,d) HRTEM image and e) profile of the interlayer distance of the pristine GDY.

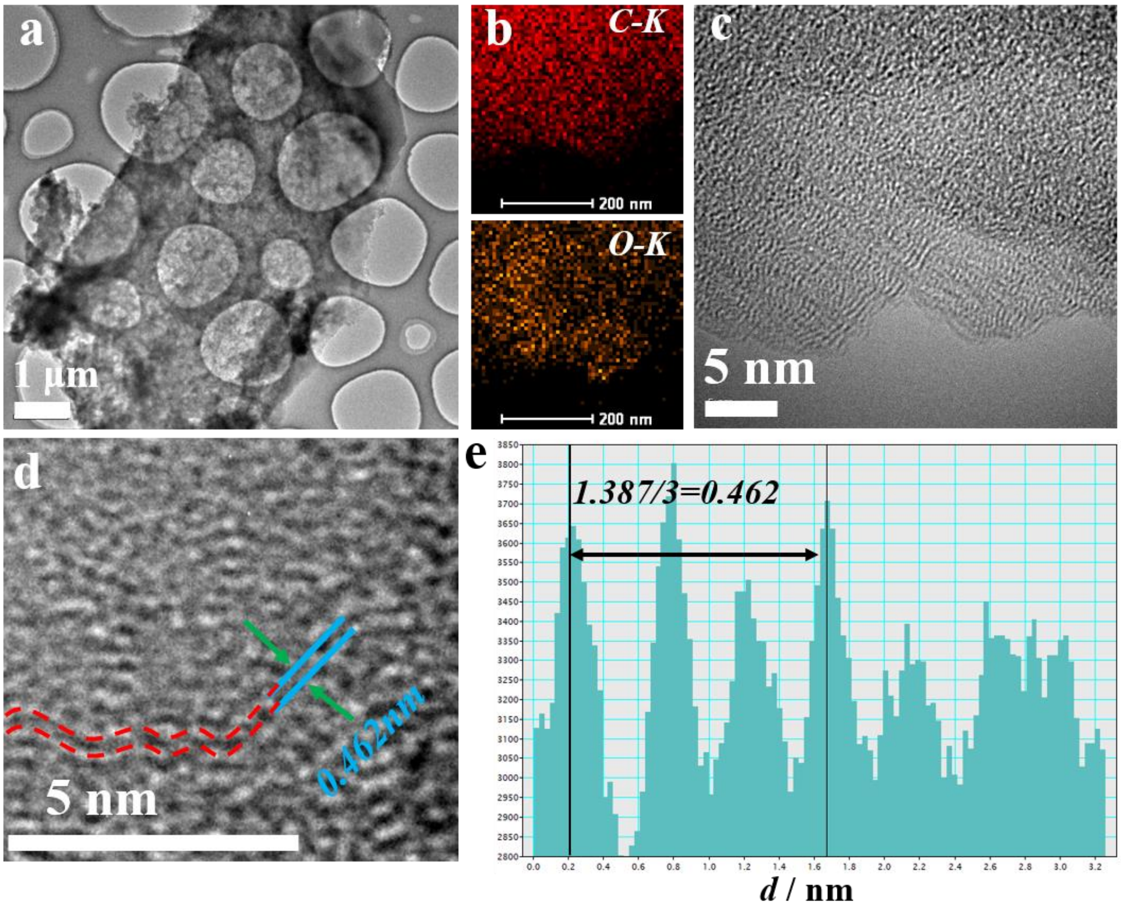

The pristine GDY exbibits the nature of well-defined 2D layered structure (**Figure S3a**), where C and O evenly distribute (**Figure S3b-c**). The interlayer spacing of GDY was measured to be 0.462 nm (**Figure S3d-e**), which is smaller than that of GDY/Fc.

Figure S4 a) N_2 adsorption/desorption isotherms and b) corresponding pore size distribution of GDY and GDY/Fc.

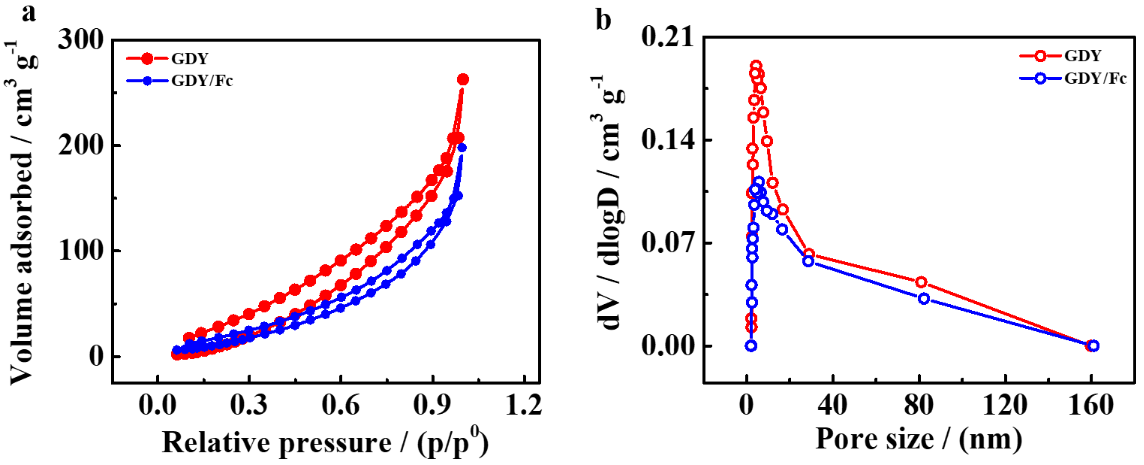

N_2 adsorption-desorption analysis (**Figure S4**) was used to determine the surface area and pore size distribution of the materials. It is calculated that the Brunauer-Emmett-Teller (BET) surface areas of GDY and GDY/Fc are 60.51 m^2/g and 53.98 m^2/g, respectively. The surface areas become smaller with the introduction of Fc, revealing the incorporation of Fc into GDY. In pore-size distribution curves, many mesoporous around 4-7 nm can be observed for GDY/Fc, which is consistent with the porous structure of GDY.

Figure S5 Digital photos of a) TEGDME with GDY/Fc after 1-day, 3-days and 7-days, b) TEGDME with soluble Fc.

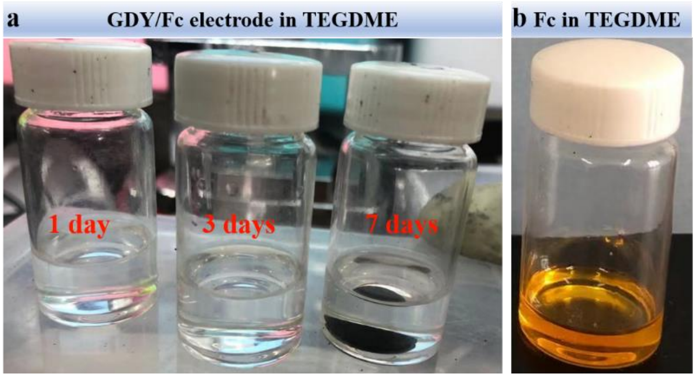

Figure S6 Chronopotentiometry curves of GDY with different soluble 1 m*M* Fc (orange), 5 m*M* Fc (violet) and 10 m*M* Fc (green).

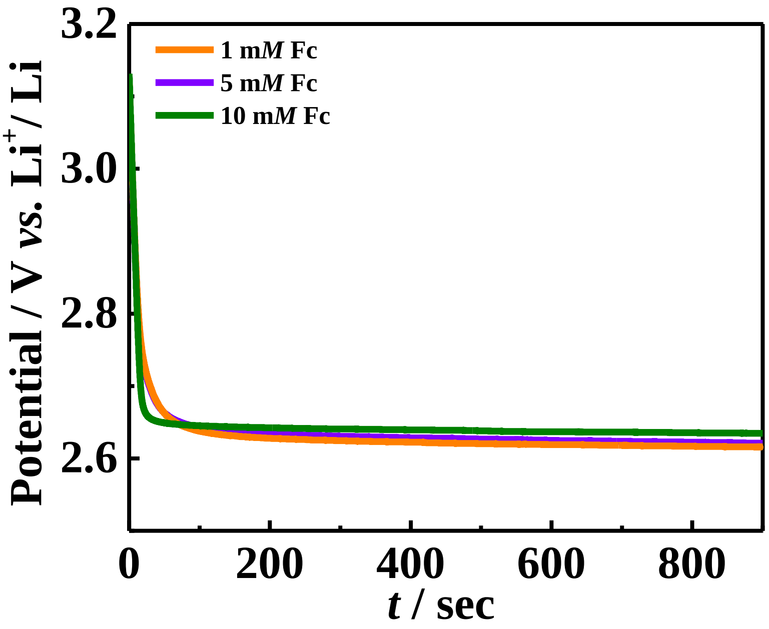

Figure S7 OER polarization curves of GDY w with different concentration soluble 1 m*M* Fc (orange), 5 m*M* Fc (violet) and 10 m*M* Fc (green).

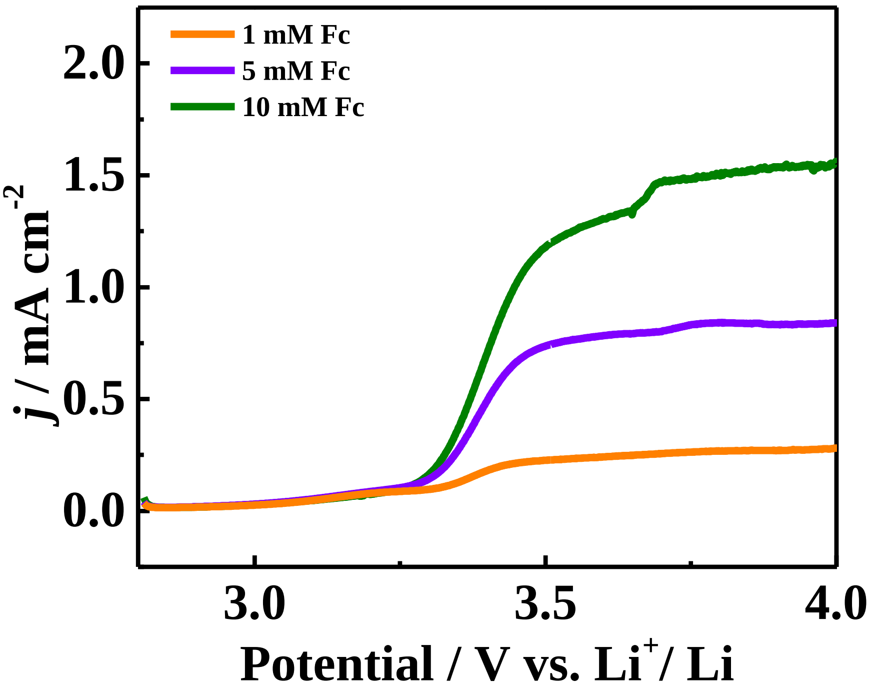

Figure S8 Chronopotentiometry curves of GDY (red), GDY/Fc (blue) and GDY with 10 m*M* Fc (green).

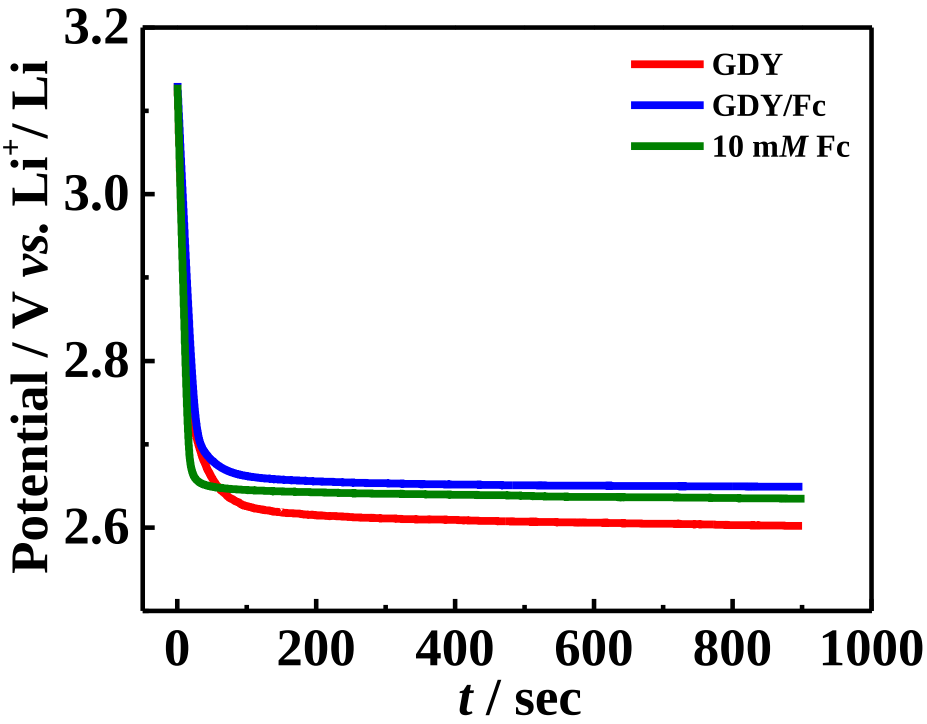

Figure S9 Galvanostatic discharge/charge curves of Li-O_2 cells with KB.

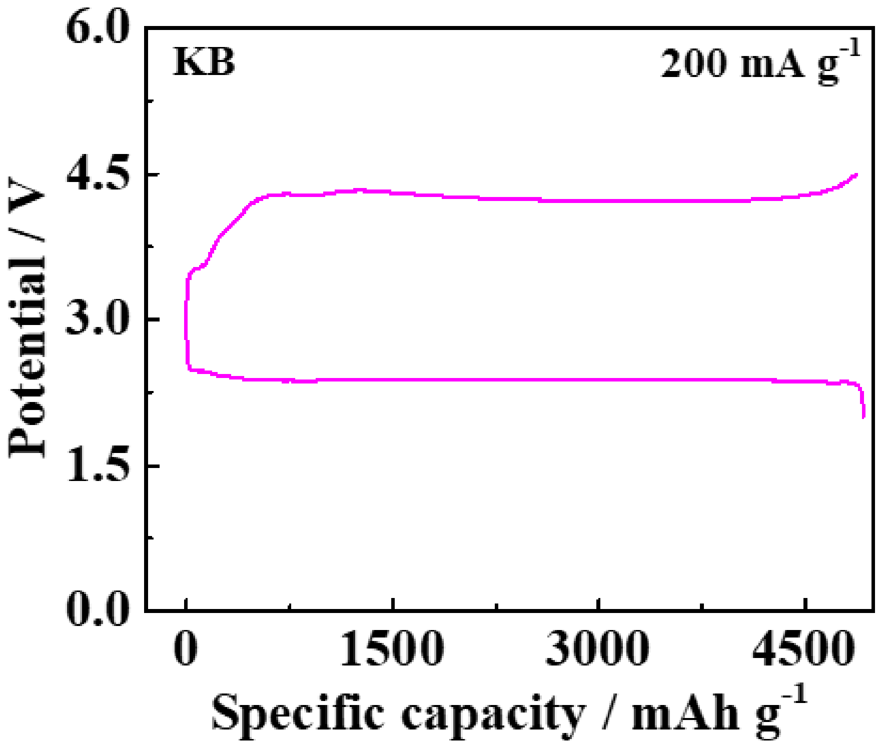

Figure S10 a) The Fe loading on the layered GDY prepared by changing the amounts of Fc addition (Fc: GDY=1:1, 5:1, 10:1) in the synthesis process. b) The corresponding galvanostatic discharge curves of LOB with different immobilized amount of Fc at a current density of 200 mA g^{-1}.

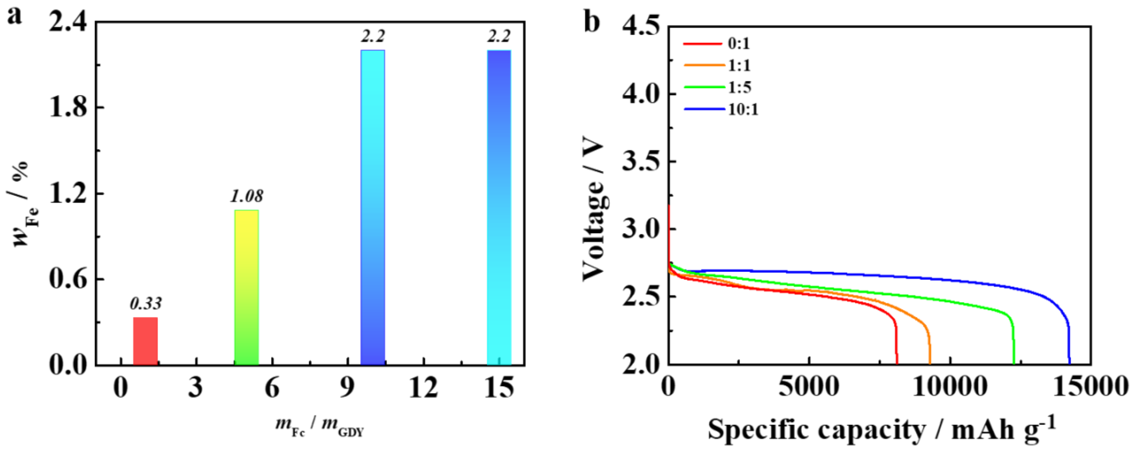

Figure S11 a) Galvanostatic discharge/charge curves of Li-O_2 cells with GDY (red line), GDY/Fc (blue line), GDY with 10 m*M* Fc at a current density of a) 400 mA g^{-1} and b) 800 mA g^{-1}.

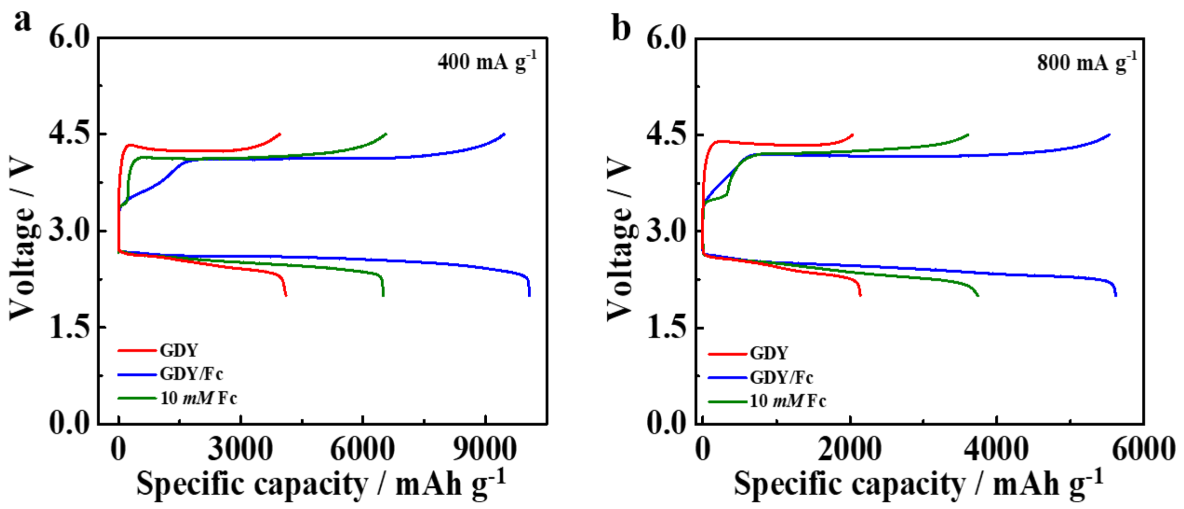

Figure S12 Galvanostatic discharge/charge curves of cells with a) GDY, b) GDY/Fc, c) GDY with 10 m*M* Fc at a current density of 500 mA g^{-1} under an upper-limit capacity of 1000 mA h g^{-1}, and d) with GDY/Fc at a current density of 200 mA g^{-1} under an upper-limit capacity of 500 mA h g^{-1}.

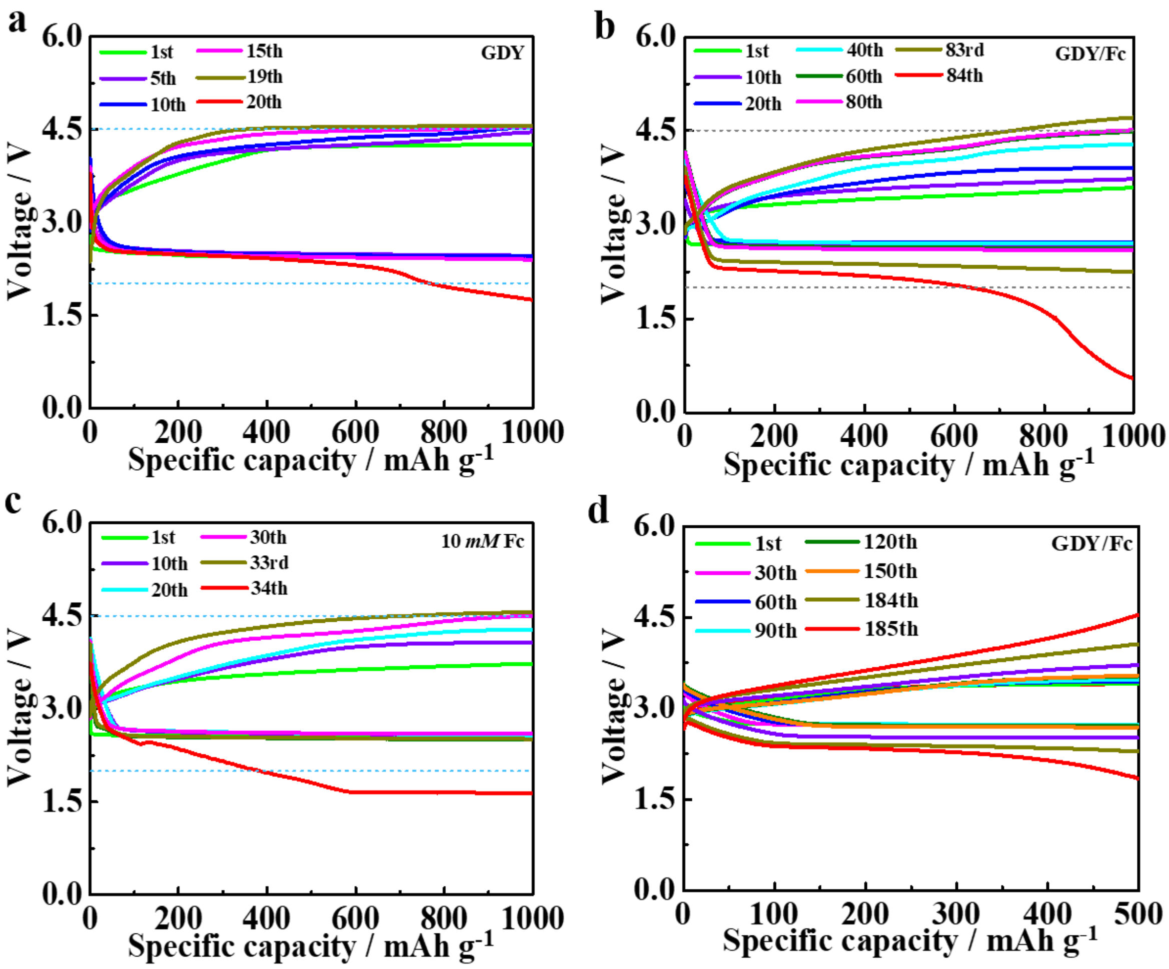

Figure S13 Cyclic performance of LOB with GDY/Fc at a current density of 2000 mA g^{-1} with a fixed capacity of 2000 mA g^{-1}.

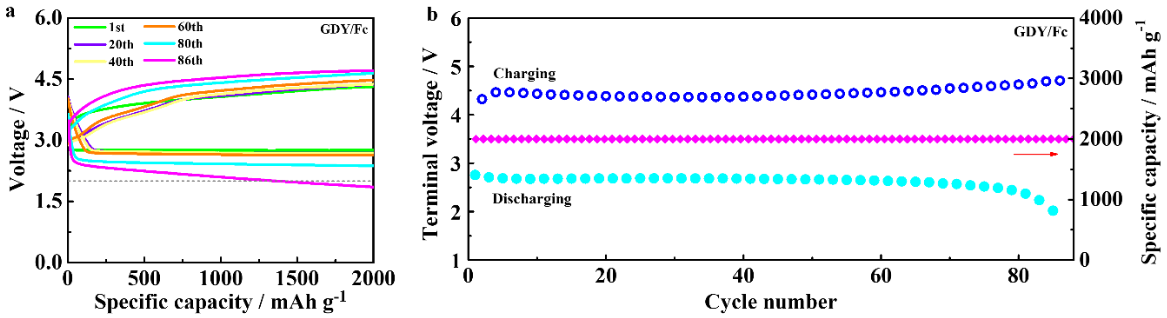

Table S1 Performance comparison for other representative published efforts.

Materials	Current Density ($mA\ g^{-1}$)	Specific Capacity ($mA\ h\ g^{-1}$)	Upper-limit Capacity ($mA\ h\ g^{-1}$)	Stability (cycles)	Reference
$Ru_{0.3}$ SAs -NC	0.02 $mA\ cm^{-2}$	13424	1000	60 cycles at 0.02 $mA\ cm^{-2}$	[22]
TiO_2	100	6568	500	40 cycles at 100 mAg^{-1}	[23]
TiC/ MWNTs	250	3841	1000	90 cycles at 250 mAg^{-1}	[24]
Co_9S_8@G/ NS-PCNF	100	8269	1000	60 cycles at 200 mAg^{-1}	[25]
Co-MOF-74	100	11350	1000	8 cycles at 250 mAg^{-1}	[26]
Pt-CNHS	200	13843	600	100 cycles at 100 mAg^{-1}	[27]
Co[Co, Fe]O_4/NG	100	13312	1000	110 cycles at 100 mAg^{-1}	[28]
**GDY/Fc**	**200**	**14231**	**500**<br>**1000**	**183 cycles at 200 mAg^{-1}**<br>**80 cycles at 500 mAg^{-1}**	**This work**

Figure S14 SEM images with different magnification of pristine GDY/Fc electrodes before discharging.

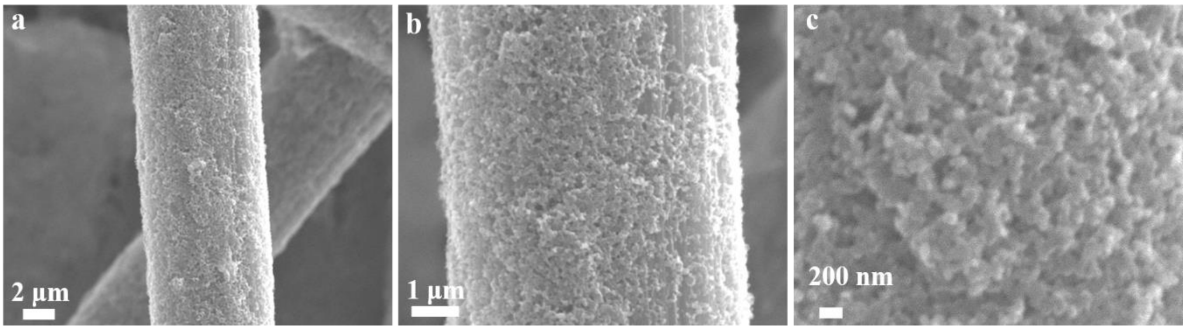

Figure S15 SEM images of the cathodes after discharged to 1000 mA h g^{-1} with a-c) GDY, b-d) GDY in the presence of 10 m*M* Fc, and g-i) GDY/Fc

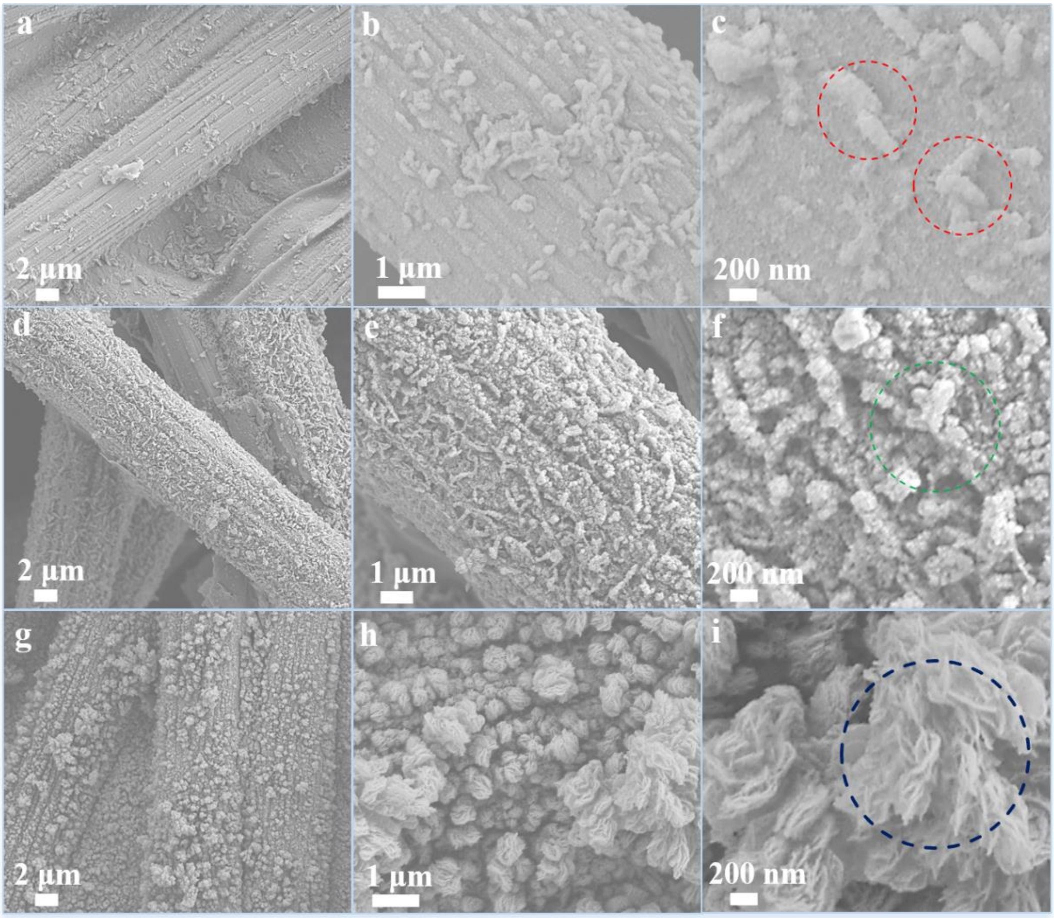

Figure S16 a) The fitting equivalent circuit and b) EIS spectra of Li-O_2 cells with b) GDY, c) GDY in the presence of 10 m*M* Fc, and d) GDY/Fc at different discharge/charge stages under a current density of 500 mA g^{-1}.

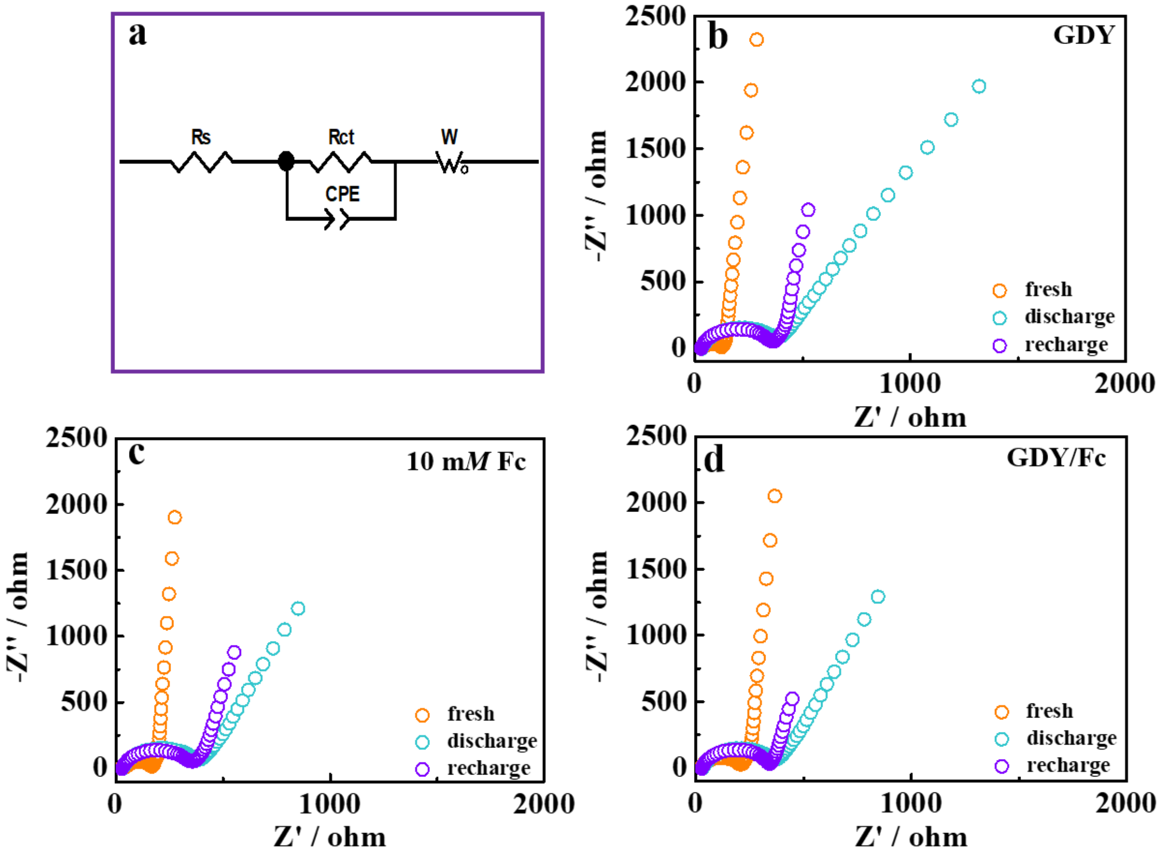

Table S2 The corresponding fitted resistance value at different discharge/charge stages.

State	***Before***	***Discharge***	***Recharge***	***ΔRecharge-Before***
Candidates	***Resistance value / ohm***			
GDY	85.1	336	298	213
GDY/Fc	167	328	287	120
10 m*M* Fc	149	309	287	138

Figure S17 a) Optimized structures of the pristine GDY/Fc and GDY/Fc after adsorbing Li_2O_2. b) Fe 2p XPS of the GDY/Fc cathode with different states. (Bottom: pristine, Top: after discharge).

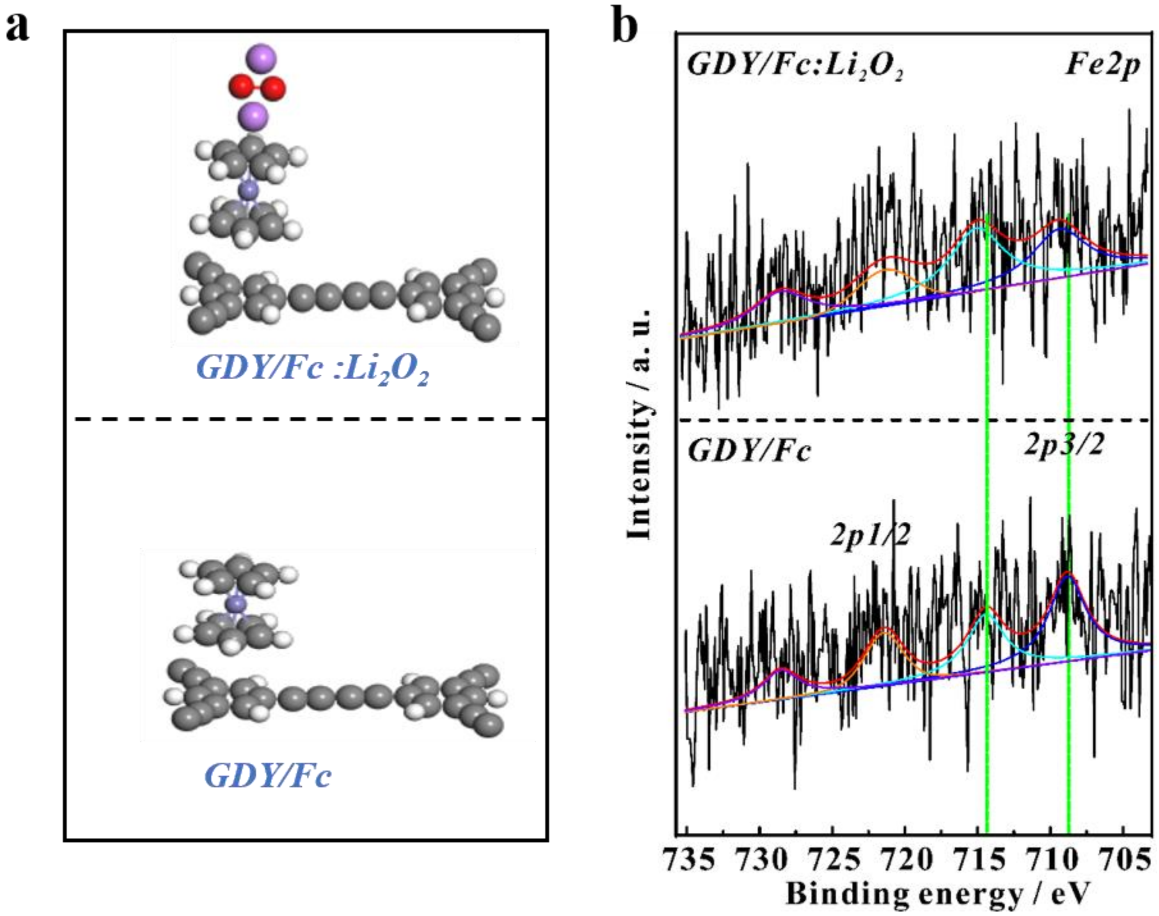

Figure S18 Raman spectra of Li-O_2 cells with a) GDY, c) GDY in the presence of 10 m*M* Fc, and d) GDY/Fc at different cycle numbers.

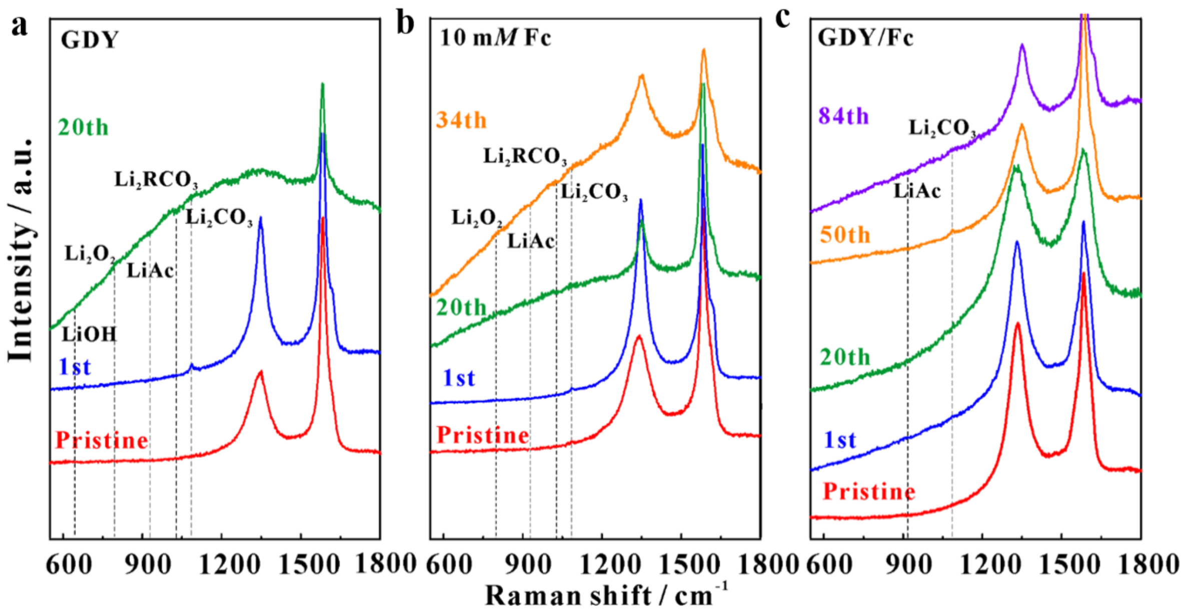

Figure S19 Fe 2p XPS spectra of GDY/Fc cathode at different cycle numbers with a)1st, b) 20th, c) 50th and d) 84th.

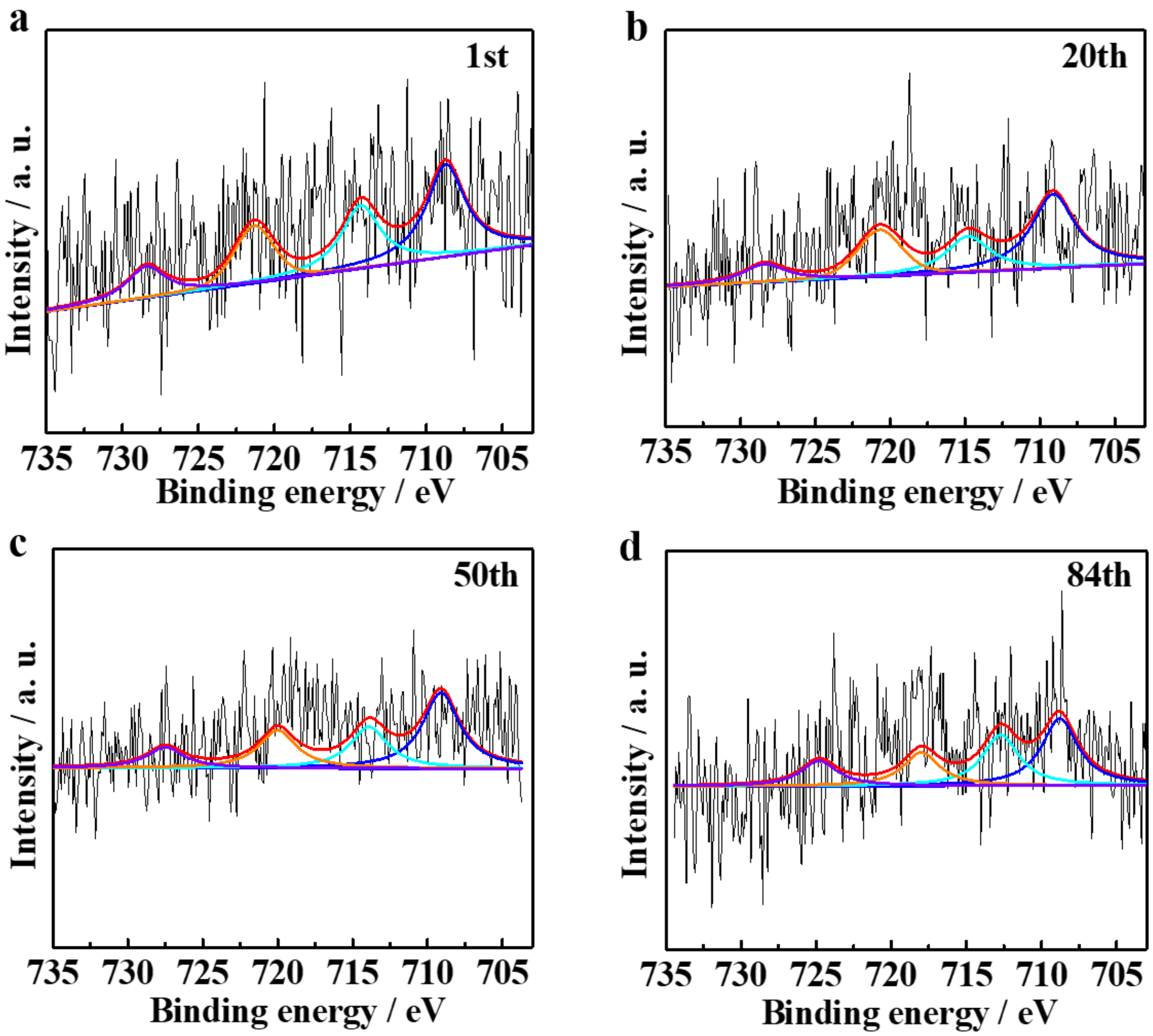

Figure S20 DEMS analysis of LOB with GDY/Fc during charging process for a) 1st cycling, b) 10th cycling and c) 100th cycling.

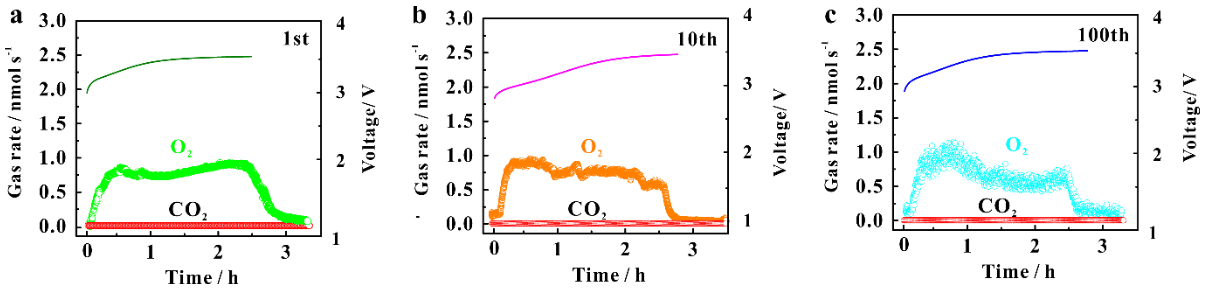

Equation S1

$$Q = I*t \qquad (1.1)$$

$$v\ (e^{-}) = Q*e^{-1} \qquad (1.2)$$

$$v\ (O_2) = GasEvol.Rate/60*NA \qquad (1.3)$$

Equation S2

Li^{+} + GDY/Fc → GDY/Fc: Li^{+} (2.1)

GDY/Fc: Li^{+} + O_2 + e^{-} → GDY/Fc: LiO_2 (2.2)

GDY/Fc: LiO_2 + Li^{+}+ e^{-}→ GDY/Fc: Li_2O_2 (2.3)

GDY/Fc: Li_2O_2 + O_2 + $2Li^{+}$+ $2e^{-}$→ GDY/Fc: $(Li_2O_2)_2$ (2.4)

Figure S21 a, b) Calculated free energy diagrams for the discharge reactions on the active surface of GDY (redline) and GDY/Fc (blue line).

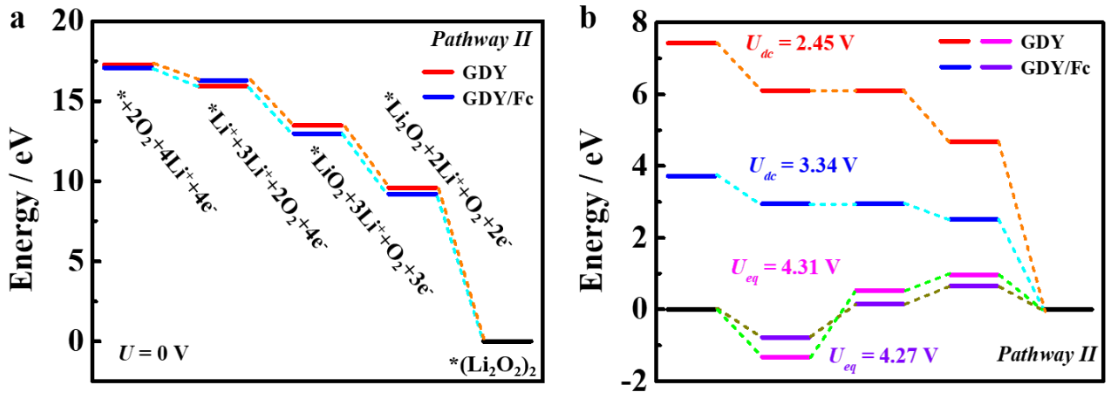

Table S3 the Δ*G* for the most endothermic step through different reaction pathways.

Reaction pathways		**ΔU / eV**	
		**GDY**	**GDY/Fc**
Pathway I	$*+ 2O_2 + 4Li^+ + 4e^- \rightarrow *O_2 + 4Li^+ + O_2 + 4e^-$	**0.58**	**0.5**
	$*O_2 + 4Li^+ + O_2 + 4e^- \rightarrow *LiO_2 + 3Li^+ + O_2 + 3e^-$		
	$*LiO_2 + 3Li^+ + O_2 + 4e^- \rightarrow *Li_2O_2 + 2Li^+ + O_2 + 2e^-$		
	$*Li_2O_2 + 2Li^+ + O_2 + 2e^- \rightarrow *(Li_2O_2)_2$		
Pathway II	$*+ 4Li^+ + 2O_2 + 4e^- \rightarrow *Li^+ + 3Li^+ + 2O_2 + 4e^-$	**1.86**	**0.93**
	$* Li^+ + 3Li^+ + 2O_2 + 4e^- \rightarrow *LiO_2 + 3Li^+ + O_2 + 3e^-$		
	$*LiO_2 + 3Li^+ + O_2 + 4e^- \rightarrow *Li_2O_2 + 2Li^+ + O_2 + 2e^-$		
	$*Li_2O_2 + 2Li^+ + O_2 + 2e^- \rightarrow *(Li_2O_2)_2$		

Figure S22 Optimized structures of GDY: O_2^-, GDY: LiO_2, GDY/Fc: O_2^- and GDY/Fc: LiO_2 and corresponding adsorption energy.

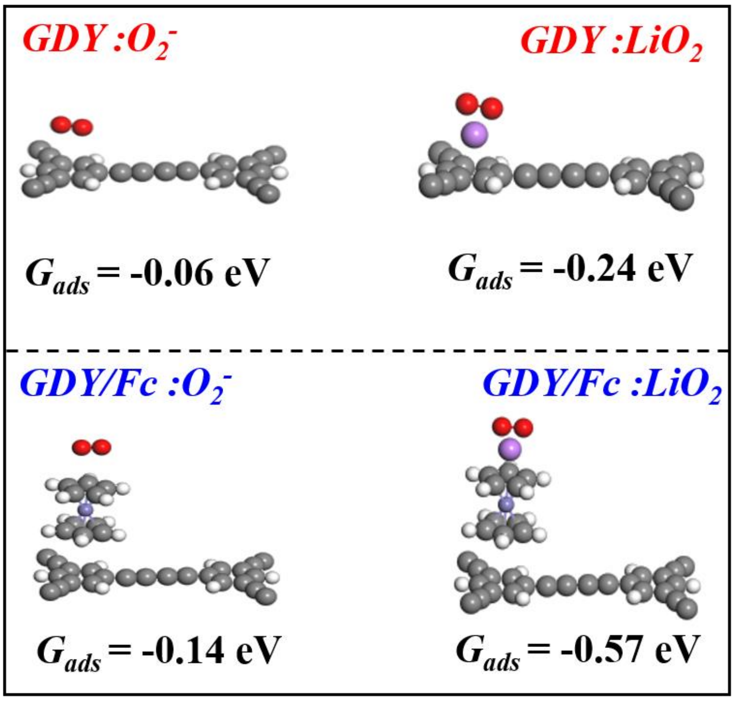

Figure S23 Digital images of a) the assembled Li-O_2 pouch cell with GDY/Fc and b) the light-emitting diode lit by the Li-O_2 battery.

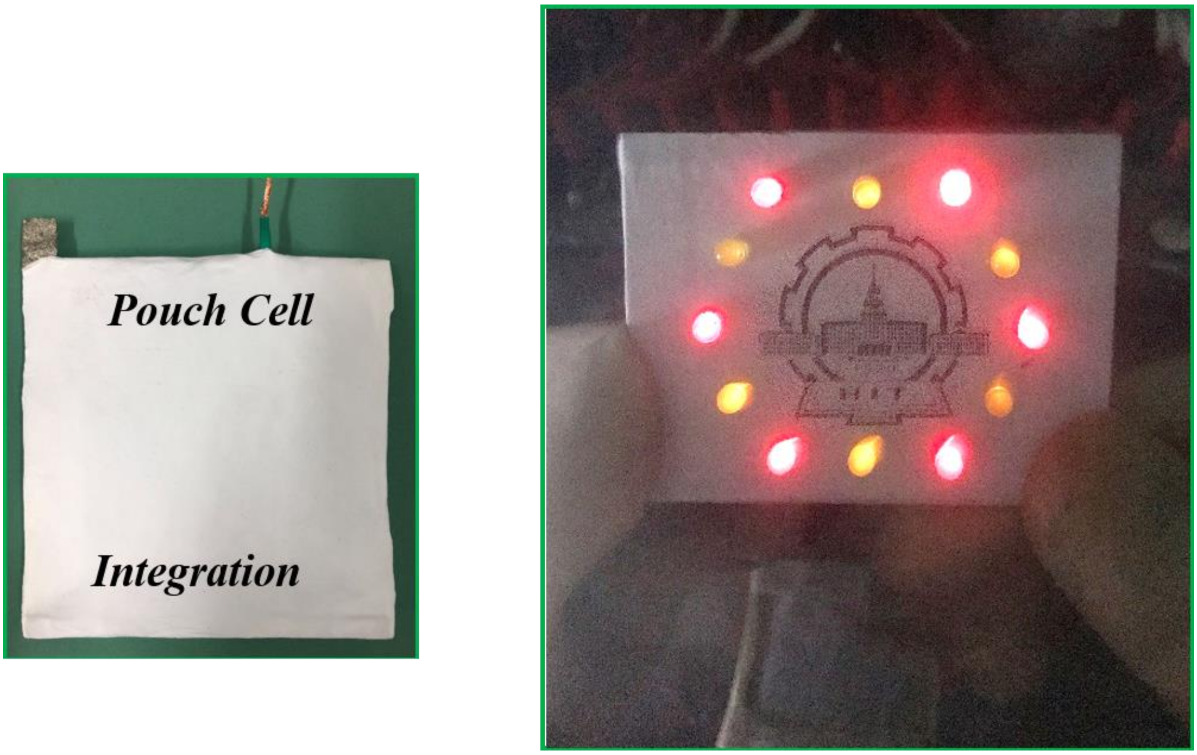

Supplement: Supplementary file 1 — Supporting Information [file ADVS-9-2103964-s001.pdf]
